# Experimental investigation into the oxidation reactivity and nanostructure of particulate matter from diesel engine fuelled with diesel/polyoxymethylene dimethyl ethers blends

**DOI:** 10.1038/srep37611

**Published:** 2016-11-23

**Authors:** Hao Yang, Xinghu Li, Yan Wang, Mingfei Mu, Xuehao Li, Guiyue Kou

**Affiliations:** 1 School of Transportation Science and Engineering, Beihang University, Beijing 100191, China

## Abstract

This paper focuses on oxidation reactivity and nanostructural characteristics of particulate matter (PM) emitted from diesel engine fuelled with different volume proportions of diesel/polyoxymethylene dimethyl ethers (PODE_n_) blends (P0, P10 and P20). PM was collected using a metal filter from the exhaust manifold. The collected PM samples were characterized using thermogravimetric analysis (TGA), scanning electron microscopy (SEM), transmission electron microscopy (TEM) and Raman spectroscopy. The TGA results indicated that the PM produced by P20 had the highest moisture and volatility contents and the fastest oxidation rate of solid carbon followed by P10 and P0 derived PM. SEM analysis showed that PM generated from P20 was looser with a lower mean value than PM emitted from P10 and P0. Quantitative analysis of high-resolution TEM images presented that fringe length was reduced along with increased separation distance and tortuosity with an increase in PODE_n_ concentration. These trends improved the oxidation reactivity. According to Raman spectroscopy data, the intensity, full width at half-maximum and intensity ratio of the bands also changed demonstrating that PM nanostructure disorder was correlated with a faster oxidation rate. The results show the use of PODE_n_ affects the oxidation reactivity and nanostructure of PM that is easier to oxidize.

Diesel engines are of importance in transportation, industry, commerce and power generation sector. Diesel engines have the advantages including efficiency, a high level of fuel economy with great torque output, high durability, reliability and low operating costs. However, emissions from diesel engines contain fine particles produced during combustion. Diesel particulate matter (PM), also known as soot, is primarily composed of carbon along with some other organic and inorganic compounds (ash), sulfur compounds, and traces of metals from unburnt fuel and lubricating oil^1^,^2^. PM is microscopic with more than 90% of PM smaller than 1 μm. PM enters human body easily via the respiratory system and can induce a variety of diseases^3^,^4^,^5^. As a consequence, emission standards for diesel PM are strictly monitored in many coutries around the world.

One prospective method to reduce PM in normal diesel engines is to use oxygenated fuel and diesel particulate filter (DPF). As a new type of oxygenated fuel, polyoxymethylene dimethyl ethers (abbreviated to PODE_n_) can effectively reduce emissions of diesel PM as PODE_n_ have no C-C bonds in their molecular structure, have a huge carbon/hydrogen ratio and a high oxygen content with a large cetane number^6^,^7^,^8^,^9^. PODE_n_ can be synthesized by polymerization reaction using methanol as a raw material^10^. There are many crude materials that can be used to produce PODE_n_, including coal and biomass. Wall-flow DPF is one of the most common technologies used in diesel vehicles to meet emissions control standards in China, U.S., Europe and elsewhere. PM regeneration is the key technology of a DPF. At present, studies on PM emitted from diesel engines fuelled with diesel/PODE_n_ blends have not frequently been reported. In this paper, oxidation reactivity and nanostructures of PM derived from diesel/PODE_n_ blends (PODE_n_ and diesel blended by volume ratio of 0/100, 10/90, 20/80; P0, P10 and P20 for short) were investigated by using thermogravimetric analysis (TGA), scanning electron microscopy (SEM), transmission electron microscopy (TEM) and Raman spectroscopy.

TGA is the most widely employed method of thermal analysis. TGA used in the analysis of PM stability, kinetic parameters and the ratio of volatile components by monitoring continuous weight loss of specimens at a controlled heating rate. Sampling is performed in the presence of an inert (i.e. He, Ar, or N_2_) and/or oxygenated environment. The main characteristic of this analytical technique is able to measure mass and PM rate of change^11^,^12^. Karin *et al*.^13^ observed that the chemical content percentage of PM can be divided into three main regions according to oxidation temperature. Moisture was vaporized at low temperature 25–200 °C, unburnt HC was oxidized at a temperature of 200–500 °C and finally, carbon inside the PM was oxidized at temperatures between 500–600 °C. Yehliu *et al*.^14^ used TGA to show that PM derived from pure biodiesel exhibited the fastest oxidation rate compared to ultra-low sulfur diesel and synthetic Fischer-Tropsch fuel. Sukjit *et al*.^15^ demonstrated that the oxidation temperature of PM decreased when using rapeseed biodiesel (RME) or oxygenated additives (butanol and castor oil methyl ester). Gill *et al*.^16^ also proved the similar results by comparing the oxidation reactivity of PM generated from conventional diesel fuel, RME-diesel and diglyme-diesel blends.

SEM has been widely applied in the observation of PM morphology. TEM has been routinely used to investigate the microstructures of diesel PM. Ishiguro *et al*.^17^ reported that a primary PM had two distinct parts; an inner core and an outer shell. The inner core had a central region with a diameter of 10 nm and consisted of several fine particles of 3 to 4 nm in diameter. The nucleus exhibited several distorted structural carbon layers. The outer shell was composed of micro-crystallites with periodic orientation of carbon sheets. Almost all of the crystallites were oriented perpendicular to the radius of the primary PM with 1 nm thick and 3.5 nm wide. Vander Wal *et al*.^18^ showed some primary soot particles had a hollow interior and an outer shell exhibiting evidence of graphitization, with a higher crystalline structure than the non-hollowed particles. Su *et al*.^19^ proposed that PM emitted from a Euro IV heavy duty diesel engine consisted of more fullerenoid- or onion-like particles agglomerated in a chain-like secondary structure and they also observed that PM with different microstructures had different oxidation reactivity.

Raman spectroscopy is an effective technique to study the structure of carbon materials as it is sensitive to structural changes and rearrangements of carbon crystallites^20^. Tuinstra and Koenig^21^ performed the earliest studies of carbonaceous materials using Raman spectroscopy in 1970. Rosen and Novakov^22^,^23^ first proved the presence of graphite-like carbon in diesel engine soot using Raman spectroscopy. The carbon in PM has a turbostratic structure between regular graphite and irregular amorphous carbon. The carbon structure and oxidation performance are both different. In the combustion process, the carbon displayed more ordered graphite-like structure which is more difficult to oxidize^24^,^25^,^26^. Oxygenated fuel can affect the carbon structure of PM. Salamanca *et al*.^27^ investigated PM produced by palm biodiesel in different proportions and showed that the degree of graphitization of PM increased with biodiesel concentration. Agedulo *et al*.^28^ compared the carbon structure of soot generated from crude palm oil, crude jatropha oil and commercial diesel fuel and found that the graphite-like structure of the soot from both biodiesels was more ordered than that of the diesel soot.

## Experimental methods

### PM sampling procedure

Experiments were performed using a single-cylinder four-stroke R180 diesel engine produced by Changchai Co., Ltd. The engine main specifications are listed in Table 1. The engine was coupled to an eddy current dynamometer providing a maximum engine power of 50 kW with a ±0.1 kW of uncertainty to control engine speed and load. Diesel fuel used in the experiments was purchased in Beijing market which meets automobile diesel fuel (V) standrad (GB 19147-2013). PODE_n_ were provided by Shandong Yuhuang Chemical Co., Ltd, which has a mass distribution of PODE_2_:PODE_3_:PODE_4_ = 2.553%:88.9%:8.48% (PODE_2-4_). Diesel fuel was used as the base fuel whilst PODE_2-4_ were used as an oxygenating additive. Sampling was performed at 1800 rpm and under 5% and 100% operating load conditions of the diesel engine. Samples were collected from an exhaust pipe 1.2 m away from the engine exhaust manifold. The direct sampling method used a metal filter. The multi-layer metal wire mesh was stacked and arranged in the exhaust pipe during the test. PM was filtered off by the metal filter to achieve similar effects to the DPF. The purpose was to obtain similar PM to that obtained under DPF actual conditions. PMs derived from different proportional diesel blends were put into sample bottles for sealing storage. Prior to each experiment, the engine was warmed up for 20 minutes.

### Analytical techniques

#### Thermogravimetric analysis

A STA-449F3 simultaneous thermogravimetric analyzer from NETZSCH was used for TGA. The heating rate of the apparatus was 0.1 to 50 °C·min^−1^, the maximum temperature was set at 1500 °C with resolution of 1 μg. In order to simulate PM conditions of pyrolysis and combustion in engine exhaust, a mixture of oxidizer gas agents with 10% O_2_ in N_2_ from Airgas and N_2_ as a purge gas was utilized for the experiments. The flow rate was limited to 100 mL/min. Specimens with an initial mass of 2 mg were heated from room temperature to 900 °C across a gradient at 10 °C/min. An inert ceramic crucible was selected to avoid the catalytic effects of a metal crucible on PM and so a controlled pyrolysis process of PM was achieved.

#### Scanning electron microscopy

The morphology of the agglomerates was obtained with SEM micrographs. To make the electrical conductivity of the samples meet the requirements for SEM observation, PM samples were previously covered with a gold film. Each of the samples was viewed in secondary electron mode using a Hitachi SU8010 microscope operated at an accelerating voltage of 15 kV with a resolution of 1.0 nm. Particle size analysis was carried out on SEM images using commercial graphic processing software package, Image-Pro Plus 6.0. The corresponding column distribution map was generated in this way.

#### Transmission electron microscopy

To investigate soot nanostructure, a Tecnai G^2^ 20 was used to capture high-resolution bright field images. The instrument was operated at 200 kV using a LaB6 filament. The applied magnification was up to 1,030,000× with a resolution of 0.24 nm. TEM samples were prepared by the following method. Firstly, a small amount of PM was ultrasonically dispersed in methanol for 30 minutes. Five drops of the solution were then placed on a 200 mesh lacey C/Cu TEM grid. Finally, after the grid and sample were air dried, TEM analysis was performed.

The nanostructure of PM was shown to be in the form of parallel or distorted carbon lamellae. Image-Pro Plus 6.0 software was employed for analyzing high-resolution TEM (HRTEM) images. In this paper, three parameters, including fringe length (*L*_a_), fringe separation (*D*_s_) and tortuosity (*T*_f_), were used to describe PM nanostructure^29^. Fringe length is a measure of the physical extent of the atomic carbon layer planes as seen in the HRTEM image. Fringe separation is the mean distance between adjacent carbon layer planes. Tortuosity is a measure of the curvature of the fringes and is defined here as the ratio of the fringe length to the distance between the two endpoints. In this study, fringe length was less than 0.4 nm and fringe separation was greater than 0.45 nm and less than 0.3 nm were discarded as artifacts.

#### Raman spectroscopy

Raman spectra of PM samples were collected via a Renishaw inVia Raman microscope. A 514.5 nm Ar ion laser was used to excite the scanning area of the PM samples. A 20 seconds exposure time and a source power of 10 mW were set to avoid altering or burning the sample. Raman spectra were recorded over a wavelength range of 100–3200 cm^−1^ with a 50× magnification objective. The spectral resolution was 1 cm^−1^. The Raman spectra of the PM consisted of first-order (800–2000 cm^−1^) and second-order (2500–3200 cm^−1^) spectra^30^. Origin software was used to perform curve fitting of the first-order spectrum and to determine the spectral parameters. Following the recommendations by Sadezky *et al*.^31^, spectra were fitted by combination of four Lorentzian functions for the G band (1580 cm^−1^), D1 band (1360 cm^−1^), D2 band (1620 cm^−1^) and D4 band (1180 cm^−1^) and one Gaussian function for the D3 band (1500 cm^−1^). Parameters including peak position, full width at half-maximum (FWHM) and the intensity of each band were derived from the decomposition. From analysis of these spectral parameters, the impact of the PODE_2-4_ blend ratio on the oxidation reactivity and nanostructure of the PM was determined.

## Results and Discussions

### Pyrolysis process analysis

**Figure 1:**
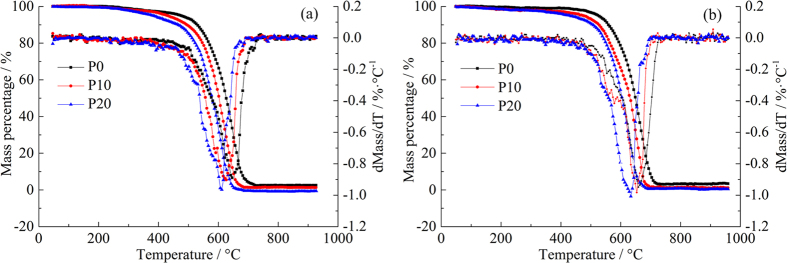
TG and DTG curves of PM samples: (**a**) low load and (**b**) high load. Operating conditions: non-isothermal experiment, initial mass of 2 mg, ramp rate at 10 °C/min, oxidizer gas (10% O_2_ in N_2_) and purge gas (N_2_) flow rate at 100 mL/min.

Pyrolysis is a basic process of thermo-chemical conversion and is the initial and associated reaction of gasification, liquefaction and combustion. Figure 1 depicts the thermogravimetric (TG) and derivative thermogravimetric (DTG) curves of PM emitted from diesel engine fuelled with P0, P10 and P20. Figure 1 shows the TG curve of moisture evaporation and volatile matter desorption at low temperature regions (60–400 °C). At low temperatures, there is no intensive chemical reaction being found. The TG curves remain relatively constant. Mass loss is insignificant at around 3%, 4% and 6% of the total mass for P0, P10 and P20 at low load, while around 1%, 3% and 5% of the total mass is loss at high load. It can be deduced that the moisture and volatile matter contents are very low in the PM samples. This may be related to the sampling method of the experiment. In the exhaust pipe where temperatures are high, most of volatile materials are found in the gas-phase and it is difficult for them to be adsorbed or coagulate onto existing PM^32^. The oxidation reaction of solid carbon in the PM takes place in the high temperature regions (400–750 °C). PM starts the oxidation process at lower temperatures with an increasing PODE_2-4_ concentration. After reaching ignition temperature, the TG curves decline rapidly. The mass loss of PM samples exceeds 95% at this stage. PM has been burnt out at 750 °C and the TG curves tend to stabilize. From Fig. 1 can also see that significant mass changes are not seen until the high temperature regions on the DTG curve. The weightlessness maximizes with increment of the PODE_2-4_ blending ratio. As PODE_2-4_ are oxygenated fuels, they may have more oxygen content in the PM which promotes oxidation. P10 and P20 show an improved oxidation rate in comparison with P0^13^.

### SEM image analysis

#### Micromorphology of PM samples

**Figure 2:**
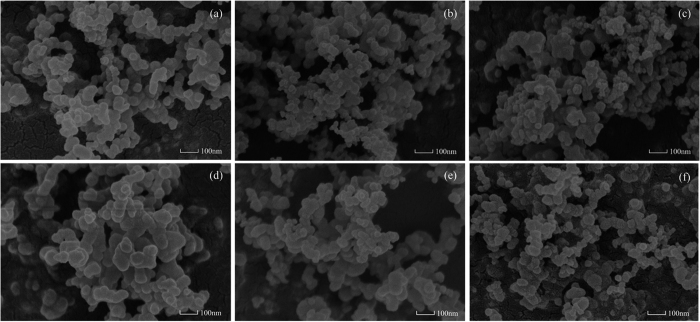
SEM images of PM samples: (**a**) P0, (**b**) P10, (**c**) P20 at low load and (**d**) P0, (**e**) P10, (**f**) P20 at high load (magnification of 100,000×).

Figure 2 reflects the SEM images of PM samples at 100,000 times magnification. As seen in Fig. 2, (1) the PM samples show certain level of agglomeration. Many quasi-spherical primary particles of different sizes, under the action of Van der Waals, electrostatic and surface adhesive forces form agglomerate particles by accumulation. (2) The PM surface is covered with a small amount of soluble organic matters as unburnt fuel is emitted into the exhaust system, and is found to adsorb on the surface of the PM. (3) PM samples generated from P0 are compactly arranged as the PODE_2-4_ blending ratio increases, the arrangement of PMs becomes looser under low and high load condition.

#### Size distribution

**Figure 3:**
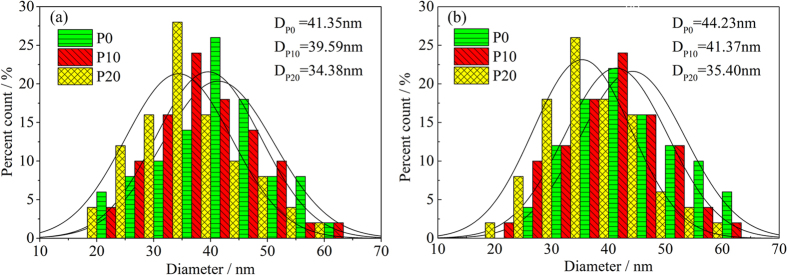
Particle size distributions and curve-fitting of PM samples: (**a**) low load and (**b**) high load.

According to Fig. 2, the size distribution of PM was determined using statistical analysis in a unit area. The mean diameters of PMs (D_P0_, D_P10_ and D_P20_) are obtained. Size distributions and the average diameter of particles at different PODE_2-4_ concentrations at low and high load are plotted in Fig. 3 which shows that particle size exhibits an approximate normal distribution in the range of 20–60 nm. With an increase in PODE_2-4_ blending ratio, particle size distribution gradually moves to the small size range and the mean diameter of the PM decreases. At low load, the average diameter of PM derived from P0 is 42.35 nm and the average diameter of PM emitted from P20 is 34.38 nm, which decreases by 18.82%. At high load, the average diameter is 44.23 nm for P0 and 34.38 nm for P20, which decreases by 19.96%. The main reason for this is that the oxygen content and cetane number are higher in the blended fuel which promotes more efficient fuel burning. It is well known that smaller and looser PM is easier to oxidatize and is advantageous for DPF regeneration.

### TEM image analysis

#### Nanomorphology of PM samples

**Figure 4:**
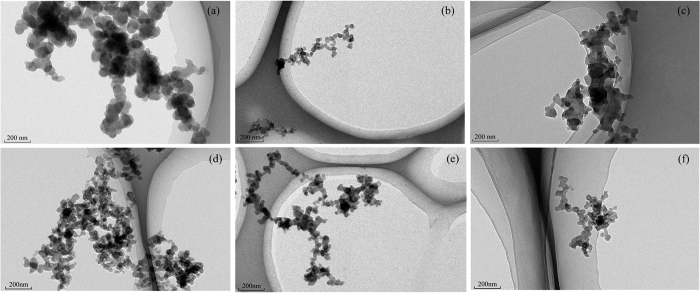
TEM images of PM samples: (**a**) P0, (**b**) P10, (**c**) P20 at low load and (**d**) P0, (**e**) P10, (**f**) P20 at high load (magnification of 50,000×).

PMs emitted from diesel engines consist of three dominant morphological types^33^: (1) individual particles which always exhibit spherulite and are the primary particles generated from diesel engine; (2) small aggregates that are formed by primary particles in the form of chains or clusters; (3) large agglomerates usually exhibiting flakes or spherules. Figure 4 shows TEM images of the PM samples. Many primary particles stack together in the darker region of TEM images making the outlines of the primary particles more obscured. As shown in Fig. 4, different degrees of density are observed. The arrangement of PMs becomes looser with increased PODE_2-4_ concentration, which is consistent with the SEM results. As for oxygenated fuel, PODE_2-4_ can make the combustion more complete and reduce the generation of PM. The probability of collision between primary particles therefore decreases, and large agglomerates are not easily formed.

#### Nanostructure parameters analysis

**Figure 5:**
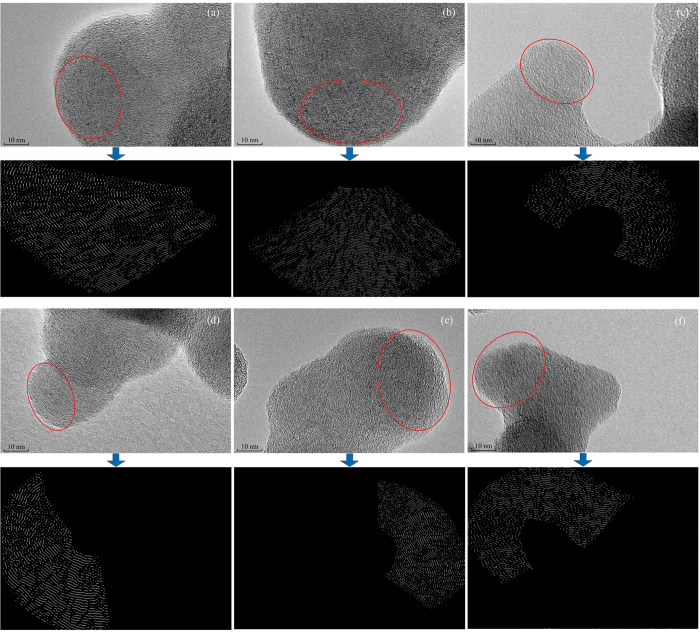
HRTEM images and the corresponding skeleton images: (**a**) P0, (**b**) P10, (**c**) P20 at low load and (**d**) P0, (**e**) P10, (**f**) P20 at high load. HRTEM images with magnified 1,000,000×. Skeleton image is obtained after image processing procedures: Choose an image region (red circle), then the three steps follow (**a**) Fourier transform; (**b**) binary conversion; (**c**) skeletonized image.

As SEM images could not completely reflect the nanostructure of primary particles, HRTEM was used in this study. HRTEM images were digitized and analyzed using image processing software to gain nanostructure parameters (*L*_a_, *D*_s_ and *T*_f_). The major steps of method are as follows: a region with clear carbon layers is selected in the HRTEM image and converted into black and white. From this binary image, a skeletonizing process was used and each fringe was changed to a one-pixel width for further statistical analysis. The original HRTEM images and the final skeleton images are depicted in Fig. 5.

The mean value and the standard deviation (sd) of each parameter are listed in Table 2. Based on Table 2, the influence of PODE_2-4_ concentration could be concluded as follows. With an increase in the PODE_2-4_ blending proportion, the average fringe length decreases gradually in the order of P0 (1.027 nm) > P10 (0.938 nm) > P20 (0.806 nm) at low load and P0 (1.041 nm) > P10 (0.950 nm) > P20 (0.819 nm) at high load. The smaller the fringe length, the higher the degree of carbon structural disorder. It was also found that disordered amorphous carbon has higher oxidation reactivity than highly ordered graphitic carbon^34^. Vander Wal and Mueller^35^ also observed that carbon atom at edge-site positions has greater activity than that in basal-plane. Small size fringe length is more likely to be oxidized because it contains a larger number of edge-site carbon atoms.

The average value of separation distance of primary particles produced from P0 is 0.383 nm at low load and 0.378 nm at high load in Table 2. Separation distance is greater with an increase of PODE_2-4_ concentration with mean value of separation distances of 0.399 and 0.410 nm at low load and 0.393 and 0.404 nm at high load, respectively, for the primary particles generated from P10 and P20. Fringe separation can enhance the oxidation reactivity of primary particles because the increase of fringe separation facilitates oxygen access to carbon layers^36^.

With an increase of PODE_2-4_ blending ratio, the mean value of tortuosity shows an increasing order of P0 (1.648) < P10 (1.667) < P20 (1.690) at low load and P0 (1.566) < P10 (1.582) < P20 (1.613) at high load. Tortuosity is attributed to the existence of 5- or 7-member rings structures in layer plane and ralates to the degree of carbon layer disorder^37^. As tortuosity increases, the proportion of the carbon layer with obvious graphite structure is reduced and the carbon layer is more easily oxidized.

#### Raman spectra analysis

**Figure 6:**
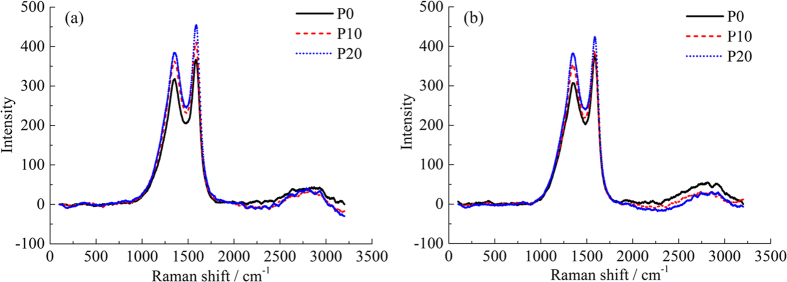
Raman spectra of PM samples: (**a**) low load and (**b**) high load. Raman spectra record over a wavelength range of 100–3200 cm^−1^. First-order is 800–2000 cm^−1^ and second-order is 2500–3200 cm^−1^.

Raman spectroscopy can be applied to acquire detailed information about the reactivity of PM by determining the structure. Differences were observed among the Raman spectra of PM samples as shown in Fig. 6. The D1 and G bands of graphite were characterized by two broad and strongly overlapping peaks near 1360 cm^−1^ and 1580 cm^−1^ for all PM samples. The G (Graphite) band corresponds to an ideal graphitic lattice vibration mode with E_2g_ symmetry at sp^2^ sites^38^. The D1 (Defect) band commonly represents the defects in the graphite structure and other disordered structures, corresponding to a graphitic lattice vibration mode with A_1g_ symmetry^39^. As seen in Fig. 6, the spectrogram does not change substantially in the range of 100–800 cm^−1^ at two load conditions. With an increase of blending ratio, the intensity of the G and D1 bands gradually increases across 800–2000 cm^−1^ showing that the vibration amplitude of carbon atoms becomes larger. In the interval between 2000–3200 cm^−1^, weak Raman vibrational peaks appear which may be related to the gap between the PMs.

#### Raman spectra fitting and parameters analysis

**Figure 7:**
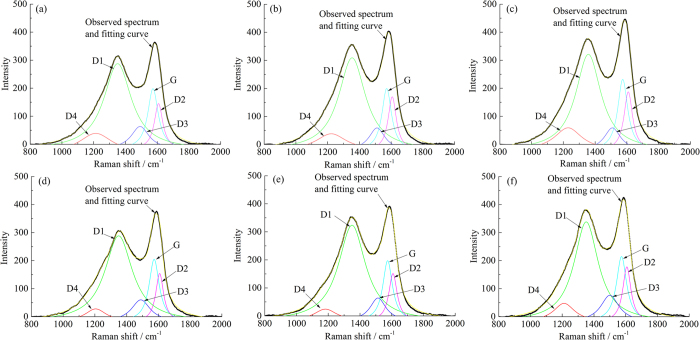
Curve-fitted for the first-order Raman spectra of PM samples: (**a**) P0, (**b**) P10, (**c**) P20 at low load and (**d**) P0, (**e**) P10, (**f**) P20 at high load. G, D1, D2, D3 and D4 bands exhibit after fitting.

All PM samples exhibited similar spectra, but the information in the graphite structure was significantly different. Figure 7 shows the fitting results of the first-order Raman spectra of PM samples. In addition to the original D1 and G bands, D2 (1620 cm^−1^), D3 (1500 cm^−1^) and D4 (1180 cm^−1^) bands are exhibited after fitting. The D2 band can be observed as a shoulder on the G band which is attributed to a vibration mode with E_2g_ symmetry involving surface graphene layers^40^. The D3 band is indicative of amorphous carbon within the PM sample including organic molecules, fragments or functional groups^41^. The intensity of the D3 band relates to organic molecules, fragments and functional group contents in the PM. The D4 band is usually a lower energy shoulder of D1 which is due to sp^2^-sp^3^ bonds or C-C and C=C stretching vibrations of polyene-like structures^42^. As shown in Fig. 7, the intensity of the D1 band is stronger compared to the G band, indicating poor order of the PM samples. The D3 and D4 bands are weaker in intensity but wider in FWHM implying amounts of amorphous carbon in the PM.

The spectra after curve-fitting can be quantitatively analyzed for revealing the changes of the bands. Raman spectral properties, such as intensity and area ratios, FWHM, and positions of the bands are an accurate representation for the degree of carbon structure order. Therefore, in the present work we focused on discussing changes in the D1 FWHM, *I*_D1_/*I*_G_ intensity ratio and *I*_D3_/*I*_G_ intensity ratio to measure the evolution of the ordered and amorphous carbon structure.

**Figure 8:**
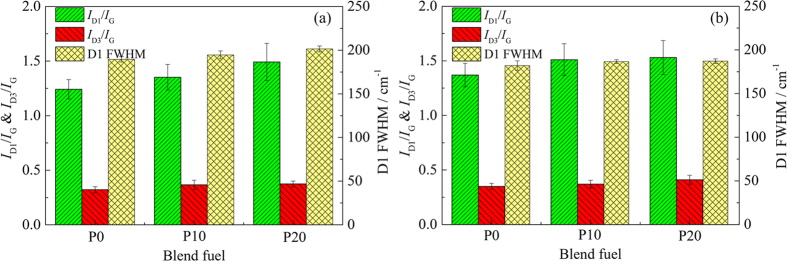
Comparison of Raman parameters of PM samples: (**a**) low load and (**b**) high load. Changes in FWHM of D1 band, *I*_D1_/*I*_G_ and *I*_D3_/*I*_G_ intensity ratio for P0, P10 and P20.

FWHM of the D1 band is related to chemical heterogeneity of the PM^43^. The wider the FWHM, the more complex components in the PM. FWHMs of P0, P10 and P20 in the D1 band are 190 ± 3 cm^−1^, 195 ± 5 cm^−1^ and 201 ± 3 cm^−1^ at low load, while at high load FWHMs of the D1 band are 182 ± 5 cm^−1^, 186 ± 2 cm^−1^ and 187 ± 3 cm^−1^, respectively, as shown in Fig. 8. The FWHM of the D1 band increases gradually with an increase of the PODE_2-4_ blending ratio. This implies that the PM contains more materials, chemical heterogeneity increases gradually and the order of graphite structures decreases.

The *I*_D1_/*I*_G_ intensity ratio usually represents the degree of graphitization and shows the oxidation activity of the PM^44^. There is an escalating trend of *I*_D1_/*I*_G_ intensity ratio with PODE_2-4_ concentration at two working conditions in Fig. 8. It can therefore be deduced that amorphous carbon content is high, and as the degree of graphitization decreases, the oxidation activity of PM improves.

As *I*_D3_/*I*_G_ intensity ratio increases, the amorphous carbon content in PM rises^45^. As seen in Fig. 8, the *I*_D3_/*I*_G_ intensity ratio increases gradually with an increment of the PODE_2-4_ blending ratio at two operating conditions indicating an increase of amorphous carbon content and a degree of carbon structural disorder. The PM is easier to oxidatize because of increased organic ingredients in the PM.

## Conclusions

This paper focuses on the impacts of the blend fuels upon the oxidation reactivity and nanostructure of PM. Diesel/PODE_2-4_ blend fuel was run on a single cylinder diesel engine with different blending ratios. The PM emitted from diesel engine was collected by direct sampling and characterized using TGA, SEM, TEM and Raman spectroscopy. The following conclusions can be drawn from analysis of the experimental work:

1. PM produced by P20 had the greatest moisture and volatile contents, and the fastest oxidation rate, followed by P10 and P0 derived PM.
2. From the SEM analysis, the PM generated from P20 exhibited the loosest and the minimum mean value than P10 and P0 which improved oxidative combustion.
3. With an increase of PODE_2-4_ concentration, fringes lengths were reduced, separation distance and tortuosity become larger. These trends could enhance the oxidation reactivity of PM.
4. With increments of the PODE_2-4_ blending ratio, changes in the Raman parameters showed that PM nanostructure disorder was correlated with a faster oxidation rate.

## Additional Information

**How to cite this article**: Yang, H. *et al*. Experimental investigation into the oxidation reactivity and nanostructure of particulate matter from diesel engine fuelled with diesel/polyoxymethylene dimethyl ethers blends. *Sci. Rep.* **6**, 37611; doi: 10.1038/srep37611 (2016).

**Publisher’s note:** Springer Nature remains neutral with regard to jurisdictional claims in published maps and institutional affiliations.

## Figures and Tables

**Table 1:** Diesel engine main specifications.

Parameter	Value
Number of cylinders	1
Rated power/kW	5.67
Rated speed/(r·min^−1^)	2600
Bore/mm × Stroke/mm	80 × 80
Displacement volume/mL	402
Compression ratio	21:1
Injection pressure/MPa	13.72 ± 0.5

**Table 2:** Nanostructure parameters (mean and sd) derived from skeleton images.

	Blend fuel	*L*_a_ (sd) (nm)	*D*_s_ (sd) (nm)	*T*_f_ (sd)
Low load	P0	1.027 (0.0673)	0.383 (0.0207)	1.648 (0.0551)
	P10	0.938 (0.0517)	0.399 (0.0243)	1.667 (0.0608)
	P20	0.806 (0.0574)	0.410 (0.0267)	1.690 (0.0549)
High load	P0	1.041 (0.0727)	0.378 (0.0319)	1.566 (0.0605)
	P10	0.950 (0.0506)	0.393 (0.0326)	1.582 (0.0653)
	P20	0.819 (0.0631)	0.404 (0.0329)	1.613 (0.0759)
